# Proceedings of the COST action BM1407 inaugural conference BEAT-PCD: translational research in primary ciliary dyskinesia - bench, bedside, and population perspectives

**DOI:** 10.1186/s12919-016-0067-0

**Published:** 2016-11-29

**Authors:** Bruna Rubbo, Laura Behan, Eleonora Dehlink, Myrofora Goutaki, Claire Hogg, Panayiotis Kouis, Claudia E. Kuehni, Philipp Latzin, Kim Nielsen, Dominic Norris, Sylvia Nyilas, Mareike Price, Jane S. Lucas, on behalf of BEAT-PCD

**Affiliations:** 1 Primary Ciliary Dyskinesia Centre, University Hospital Southampton NHS Foundation Trust, Southampton, UK; 2 University of Southampton Faculty of Medicine, Academic Unit of Clinical and Experimental Medicine, Southampton, UK; 3 Primary Ciliary Dyskinesia Centre, Department of Paediatrics, Royal Brompton and Harefield Foundation Trust, London, UK; 4 Institute of Social and Preventive Medicine, University of Bern, Bern, Switzerland; 5 Paediatric Respiratory Medicine, Children’s University Hospital of Bern, University of Bern, Bern, Switzerland; 6 Cyprus International Institute for Environmental & Public Health, Cyprus University of Technology, Limassol, Cyprus; 7 Danish PCD & chILD Centre, CF Centre Copenhagen, Paediatric Pulmonary Service, Department of Paediatrics and Adolescent Medicine, Copenhagen University Hospital, Rigshospitalet, Denmark; 8 MRC Harwell, Harwell Campus, Oxfordshire, UK; 9 Department of Paediatric Pulmonology, University Children’s Hospital Basel (UKBB), Basel, Switzerland; 10 Department of Paediatric Pneumology, Allergology and Neonatology, Hannover Medical School, Hannover, Germany; 11 Biomedical Research in Endstage and Obstructive Lung Disease Hannover (BREATH), Member of the German Center for Lung Research, Hannover, Germany; 12 Faculty of Medicine Mail Point 803, University Hospital Southampton, Southampton, SO16 6YD UK

## Abstract

Primary ciliary dyskinesia (PCD) is a rare heterogenous condition that causes progressive suppurative lung disease, chronic rhinosinusitis, chronic otitis media, infertility and abnormal situs. ‘Better Experimental Approaches to Treat Primary Ciliary Dyskinesia’ (BEAT-PCD) is a network of scientists and clinicians coordinating research from basic science through to clinical care with the intention of developing treatments and diagnostics that lead to improved long-term outcomes for patients. BEAT-PCD activities are supported by EU Framework Programme Horizon 2020 funded COST Action (BM1407). The Inaugural Conference of BEAT-PCD was held in December 2015 in Southampton, UK. The conference attracted ninety-six scientists, clinicians, allied health professionals, industrial partners and patient representatives from twenty countries. We aimed to identify the needs for PCD research and clinical care, particularly focussing on basic science, epidemiology, diagnostic testing, clinical management and clinical trials. The multidisciplinary conference provided an interactive platform for exchanging ideas through a program of lectures, poster presentations, breakout sessions and workshops. This allowed us to develop plans for collaborative studies. In this report, we summarize the meeting, highlight developments, and discuss open questions thereby documenting ongoing developments in the field of PCD research.

## Introduction

PCD is a rare heterogeneous disorder characterized by impaired mucociliary clearance due to abnormal ciliary function, which is usually but not always associated with abnormal ciliary ultrastructure [1, 2]. Clinical manifestations are caused by impaired mucociliary clearance and include recurrent lower and upper respiratory tract symptoms which present soon after birth. Neonatal symptoms range in severity from mild transient tachypnoea to significant respiratory failure requiring prolonged respiratory support [3]. Recent data suggests that PCD has a progressive, and potentially severe long-term course of lower airway disease [4] with recurrent infections leading to bronchiectasis and impaired lung function. Male infertility is common since sperm flagella have a similar ultrastructure to cilia, whereas the incidence of female infertility and of ectopic pregnancy is uncertain but might be explained by immotile fallopian tube cilia [5]. Motile embryonic nodal cilia establish left-right asymmetry [6] and nearly half of PCD patients exhibit situs inversus [7] and 6–12% have heterotaxic syndromes (abnormal arrangement across the left-right axis of the body) which can be associated with complex congenital cardiac defects [7–9]. PCD is a genetically heterogeneous disorder, typically caused by an autosomal recessive mode of inheritance (more than 30 genes identified to date); diagnostic and molecular features differ according to the specific gene and mutations. Diagnosis is currently based on combination testing, which normally includes nasal nitric oxide (nNO) measurements, ciliary beat frequency (CBF) and pattern (CBP) using high-speed video microscopy analysis (HSVMA), ultrastructural defects using transmission electron microscopy (TEM), and genetic testing [10].

Data is lacking on genetic and environmental determinants of clinical phenotype, severity, or long-term prognosis. Reported prevalence of PCD varies across Europe reflecting true variability as well as differences in access to diagnostic facilities [10]. Prevalence is estimated 1:2000–1:40,000, with true prevalence probably 1:10,000 or higher [11]. This reflects a significant disease burden, causing progressive disease in 74,000 Europeans. A quarter of adult PCD patients in USA exhibit severe lung disease requiring long term oxygen or lung transplantation [2] highlighting the need for treatments to limit disease progression. Hampering the trajectory of respiratory decline would have positive implications for health care expenditure and associated benefits to individuals, carers and society. As for other rare diseases the evidence base for PCD is sparse and there has been little clinical or translational research, with treatment strategies inappropriately extrapolated from other diseases [10, 12, 13] *e.g.,* treatments for lung manifestations are derived from cystic fibrosis (CF) guidelines despite different pathophysiology.

Over recent years advances made in the field of PCD have been attained through collaborations of clinicians on the one hand, and scientists on the other. Several international initiatives have stimulated these advances including the North American Genetic Disorders of Mucociliary Clearance Consortium (GDMCC) [8, 14–16], two network European Respiratory Society (ERS) Task Forces [10, 11, 17, 18] and European FP7-funded BESTCILIA [19–21]. To maintain this momentum and build on successes of previous collaborations, there was a need for a network to bring clinicians and scientists together. BEAT-PCD (http://www.beatpcd.org/) is a Europe-led collaboration supported by EU- Framework Horizon 2020 funded COST Action (BM1407). The international network includes experts from multidisciplinary clinical specialties (e.g., paediatric & adult pulmonology, ENT, physiotherapy, fertility) motivated for collaborative research with scientists from diverse backgrounds (e.g., genetics, imaging, cell biology, microbiology, bioinformatics) and different countries. BEAT-PCD aims to facilitate PCD-related research to identify mechanisms, study disease patterns and progression, define outcome measures, improve clinical management and identify high priority therapies. The Action aims to act as a platform throughout the process, from preclinical studies to clinical trials.

**Fig. 1:**
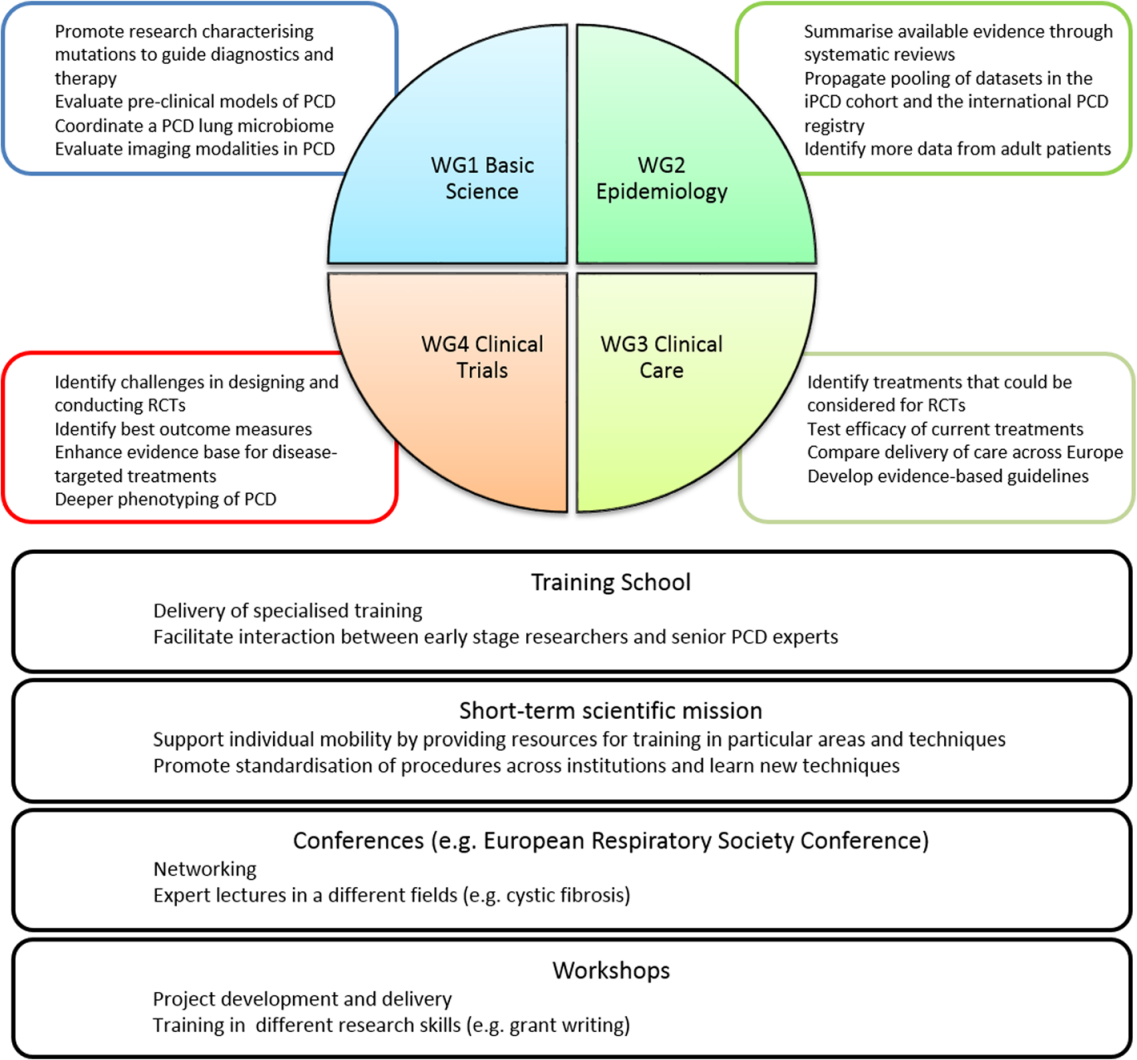
Diagram of BEAT-PCD Workgroups (WG), overall aims and training opportunities

The activities of BEAT-PCD are coordinated through four highly integrated Workgroups: Basic Science, Epidemiology, Clinical Care and Clinical Trials (Fig. 1). BEAT-PCD’s overarching mission is to provide a platform to encourage networking, to advance research underpinning diagnosis, and advance management of PCD; in particular to facilitate two-way communication between basic science and the PCD clinical community, helping these two communities to come together in highlighting important gaps in basic knowledge, unmet clinical needs and ultimately to move novel discoveries from the bench to the clinic. Major areas of interest were highlighted during the conference which stimulated discussion and brought together perspectives from basic scientists, clinicians, researchers and most importantly, patient representatives.

BEAT-PCD’s Inaugural Conference was held in Southampton, UK in December 2015. We aimed to identify the research and clinical needs pertinent to diagnosing and treating patients with PCD. In particular, we wanted to bring scientists and clinicians together to discuss collaborative approaches to expedite drug development through to clinical trials. We wanted to identify the gaps in our knowledge around basic science, epidemiology, clinical care and clinical trials, and identify the gaps that might be answered with existing data. We additionally planned to identify approaches and collaborations for prospective data collection through new studies. The conference also aimed to develop detailed plans for future studies and projects in small groups, including concrete ideas for study design, potential collaborators and funding.

This manuscript summarises the outcomes of the 3-day Inaugural Conference, which included lectures, break-out discussion groups, workshops and a poster session. Aims, projects and major areas of interest from each workgroup are addressed in separate sections. Highlights from some of the lectures are captured throughout the manuscript in boxes, and a large selection of poster titles and authors are displayed in a table.

## Workgroup 1: Basic science

The basic science workgroup (WG1) aims to develop both a research network and an infrastructure to enable the sharing of samples, data and state-of-the-art knowledge between scientists studying cilia biology and PCD pathology.

Major areas of interest for collaboration identified by WG1 include:

- Characterization of PCD causing mutations;
- Description of pre-clinical PCD models and how such models can be utilized in developing therapies;
- Understanding the airway microbiome in PCD patients; and
- Application of imaging technologies to facilitate PCD research and patient diagnostics.

**Fig. 2:**
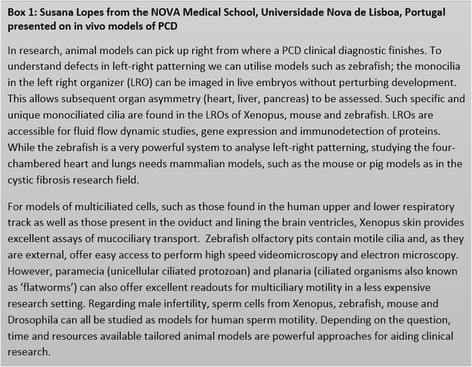
Susana Lopes from the Nova Medical School, Universidade Nova de Lisboa, Portugal presented on in vivo models of PCD

Model organisms have long been used to study motile cilia. For example, mutant studies in the *biflagellate* green alga *Chlamydomonas reinhardtii* date back many decades [22]. Current models of ciliary dyskinesia were presented at the conference (Fig. 2). In order to utilise these as pre-clinical models, one of the first goals of WG1 is to summarize the current knowledge regarding their application in PCD research in a state-of-the-art manuscript. This will be followed by the evaluation and subsequent dissemination of existing and newly developed protocols and standard operation procedures and ultimately by establishing an online platform and agreement templates that allow the simplified sharing of cells, tissue and tools between participating researchers.

Mutations in more than thirty genes are known to underlie PCD [23], raising the likelihood that personalised therapies will need to be developed. Such treatments, targeting specific genetic and/or cellular defects, seem likely to include gene therapy. Recent proof-of-principal studies have demonstrated the rescue of ciliary beating in air-liquid interface (ALI) cultures from PCD patients. Cells with mutations on *DNAI1* (explaining up to 14% of PCD cases [23]) have been rescued by a virally delivered mini-gene [24], and cells with a *DNAH11* mutation (responsible for approximately 20% of PCD with normal ultrastructure [23]) have been corrected using gene editing by a virally encoded transcription activator like effector nucleases (TALEN) [25]. Such approaches have yet to be successfully transferred into animal models, the next step on the road to human trials. The mouse is the only genetic model with lungs and while modelling aspects of PCD [26], current mouse models do not fully recapitulate human pathogenic mutations. Another objective of WG1 is therefore to foster discussion about the mutations that should be a priority for genetic modelling, based on current translational and clinical needs. Through the WG1 network, a research consortium will need to be formed to apply for funding from national and international sources to enable development of new animal PCD mutants.

**Fig. 3:**
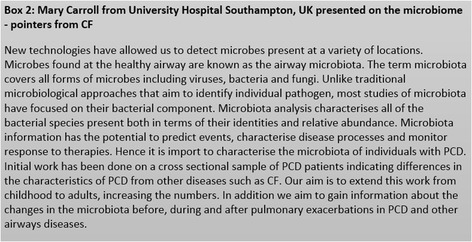
Mary Carroll from University Hospital Southampton, UK presented on the microbiome-pointers from CF

Microbiomes have an important role in human physiology and health [27]. However, there is a lack of published evidence regarding the role of pathogens in the severity and progression of PCD. Only recently have molecular techniques been utilized to identify specific clones of *Pseudomonas Aeruginosa* from chronically colonized PCD patients [28]. This meeting brought together a group of scientists and clinicians with interests in the pulmonary microbiome and PCD. Following presentation (Fig. 3) and discussion of the implications of how changes in the microbiome might influence PCD progression (and to what extent such changes either follow or drive PCD pathogenesis), a framework was described for an initial pilot study, examining the microbiota, virome and fungi composition in a cohort of PCD patients from across Europe.

**Fig. 4:**
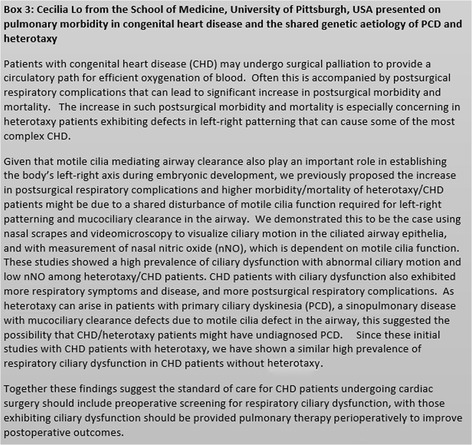
Cecilia Lo from the School of Medicine, University of Pittsburgh, USA presented on pulmonary morbidity in congenital heart disease and the shared genetic aetiology of PCD and heterotaxy

An important area of PCD research is defining the boundaries of what is considered to be PCD. There is a broad spectrum of cilia-related disorders, comprising a highly heterogeneous group of phenotypically and genetically different diseases; PCD refers to a specific syndrome associated with defects of motile cilia but our definition of PCD is developing in line with increasing knowledge. Understanding the genetic basis of disorders that can be associated with PCD (e.g., heterotaxy) may provide a deeper understanding of classical presentations of PCD and might challenge our current definition of PCD (Fig. 4).

Imaging has become central to modern science and medicine, playing key roles in both the study of cilia biology and the diagnosis of PCD. At this meeting the use of High-Speed Video Microscopy to analyse ciliary beating for diagnosis was discussed and a need for standardisation of methods and quantitative beat pattern analysis was identified since qualitative analysis is inherently subjective, even when performed by expert microscopists. The role of computer aided analysis was discussed; a number of talks and posters presented during the meeting providing evidence for these discussions. The group established a plan to share archived video data (from patient and control samples) for blind computational analysis; this can subsequently be compared to the original expert qualitative analysis.

The concept of a database, that could incorporate genomic, phenotypic (ciliary motility and ultrastructural findings) and clinical data (respiratory and other systems) along with animal model data was debated. This could also include links to reference databases such as PubMed, Scopus and could provide an open “one stop” source of information for PCD researchers.

## Work group 2: Epidemiology

The epidemiology workgroup (WG2) aims to improve our understanding of the epidemiology of PCD. It will make use of all existing data from PCD patients to gain essential knowledge on the clinical presentation and natural history of the disease and on predictors of disease progression. This will be a basis for planning future intervention studies but also for standardising and improving future prospective data collection.

Major areas of interest for collaboration identified by WG2 include:

- Systematic reviews summarising the available evidence; and
- Development of large standardised collaborative studies by pooling national and regional datasets into large standardised collaborative studies. This includes the international (iPCD) cohort, a retrospective cohort of available datasets, and the prospective international PCD registry. Particular emphasis will be given to data derived from recruitment of adult patients.

**Fig. 5:**
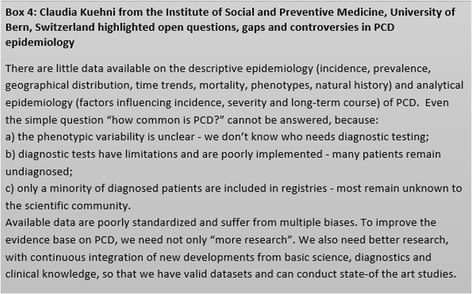
Claudia Kuehni from the Institute of Social and Preventive Medicine, University of Bern, Switzerland highlighted open questions, gaps and controversies in PCD epidemiology

Few publications are available on the clinical epidemiology of PCD and most come from case reports or small clinical case series. Many basic questions are unanswered (Fig. 5). A recent systematic review found only 52 publications that contained clinical information on PCD [9]. Identified studies were small, with a mean of 38 patients per study. Results were rarely age-stratified, outcomes not standardised, and study populations highly selected as they came from specialised clinics (e.g., pulmonary, ENT or fertility). Few studies had longitudinal data, such as changes of lung function over time [29, 30], and most had been conducted by paediatricians, resulting in little data from adult patients. However, while early reports describing children with PCD suggested a relative benign course, recent data from adult patients demonstrates severe lung disease with chronic Pseudomonas infection and respiratory failure in many patients [31].

**Fig. 6:**
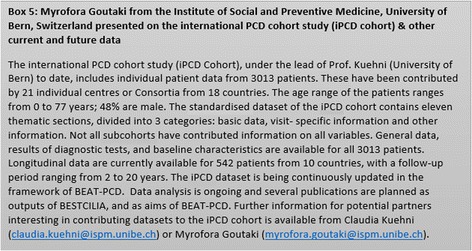
Myrofora Goutaki from the Institute of Social and Preventive Medicine, University of Bern, Switzerland presented on the international PCD cohort study (iPCD cohort) & other current and future data
- A prospective international PCD registry, also funded within BESTCILIA, provides a valuable tool for prospective data collection and future research [19].

Available resources to study the epidemiology of PCD include the two large collaborative datasets that have been compiled in the framework of the EU-funded FP7 project BESTCILIA:

- A retrospective international cohort study (iPCD cohort) has been constructed by identifying existing datasets of PCD patients, standardising and assembling them a retrospective cohort (Fig. 6). WG2 aims to maintain, expand and enrich, clean and analyse the data of the iPCD Cohort. Some contributing partners are adding new patients to their datasets; others add repeated measurements (longitudinal data) to patients that have already been included. Additional groups have expressed an interest to contribute. A methodological paper describing the development, dataset and first results of the iPCD Cohort has been submitted [32].

WG2 aims to develop these two resources further by inviting more countries and more centres to contribute data. Particular emphasis will be on recruiting more adult physicians and including more data on adult patients. The registry will establish a steering committee, which will be responsible for managing the registry in the future, and a framework of agreements regulating how these data can be used by the scientific community. In this inaugural meeting, we discussed a list of scientific publications based on these datasets, which will describe clinical presentation and long-term prognosis of PCD, such as body growth, lung growth, lung function decline with age, development of bronchiectasis and respiratory insufficiency, and changes of clinical presentation throughout lifetime. First analyses of the iPCD dataset will focus on body growth and lung function. Participants agreed on the importance to apply for funding from national and international sources to support the exploitation of the collected data.

The need for standardised clinical data on symptoms and signs, captured through structured clinical proformas, was an important topic to arise from the discussions. While standardised data are usually available on results of diagnostics tests, lung function, microbiology and imaging, the data on the spectrum, frequency and severity of symptoms (from the patient history), and physical signs (from the clinical examination) lack details and are so poorly standardised within and across clinics, that is difficult, if not impossible to analyse such data in collaborative studies. Currently PCD centres collect such information in an open format, or use forms derived from other respiratory diseases (*e.g.,* cystic fibrosis). The few clinical proformas that are PCD-specific forms are not standardised between centres. An important goal of WG2 is therefore to develop standardised clinical proformas for PCD outpatient clinics. These should enable standardised monitoring of clinical symptoms and signs at diagnosis and during follow-up, for different age-groups. The development of these proformas is a main focus for the next 2 years and will happen in collaboration with WG4. As a first step we are collecting all PCD proformas that are currently in use. These will be used to draft a standardised form, which will be refined in a Delphi process among paediatric and adult physicians caring for PCD patients.

Screening tools for referral of patients to diagnostic testing were discussed at some length. These can help to decide which patients with chronic cough and rhinitis should be referred to PCD reference centres for diagnostic testing. One prediction tool (PICADAR) has been developed and validated in two PCD centres from the UK, but needs further validation in other countries, other health care settings and different age groups of patients [33].

## Work group 3: Clinical care

The clinical care workgroup (WG3) aims to identify current and develop future strategies for diagnosis, management and delivery of care in PCD across Europe and globally. The mission is to bring together clinicians from multi-disciplinary backgrounds to work with patient representatives to improve management strategies.

Major areas of interest for collaboration identified by WG3 include:

- Identify treatments that could be considered for clinical trials;
- Identify treatments already in use that might be suitable for efficacy testing in (*e.g.,* use of antibiotics and sinus washes);
- Investigate differences in delivery of care across Europe and identify features associated with good clinical outcome; identify priority areas for research through surveys and systematic literature reviews; and
- Develop evidence based guidelines.

*Pseudomonas aeruginosa* (PsA) is an opportunistic pathogen that frequently causes chronic infections in the upper and lower airways of both PCD and cystic fibrosis (CF) patients [28, 34]. PCD patients are usually colonised in childhood, where up to one third exhibited at least one positive sample during a 6 year follow-up but only 2–5% became chronically infected [30]. Despite intensive antibiotic therapy, prevention of chronic infection is rarely achieved. Thus, approximately 30% of Danish PCD patients are chronically infected in early adulthood [28]. Chronic infection with PsA has been shown to be associated with lower lung function [31] and increasing age [7, 28]. In contrast to CF, it was recently shown that chronic infection was cleared for a minimum of 1 year in 69% of patients following treatment with a course of inhaled and oral antibiotics with an additional course of intravenous antibiotics every 3 months [28].

**Fig. 7:**
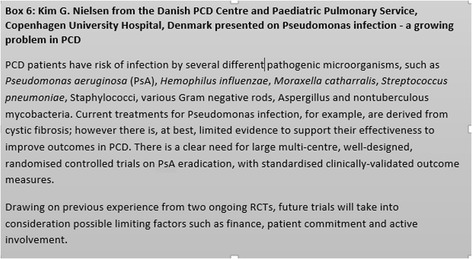
Kim G. Nielsen from the Danish PCD Centre and Paediatric Pulmonary Service, Copenhagen University Hospital, Denmark presented on Pseudomonas infection - a growing problem in PCD

A core goal in PCD management is the prompt treatment of new infections through eradication of PsA. Eradication protocols were recently recommended by experts [35], and are based on those used in CF. Experts also recommend treatment of chronic PsA infection, often based on use of nebulized antibiotics [35] and some PCD specialist centres advocate the use of long-term prophylactic antibiotics. The growing problem of PsA infection in PCD was presented (Fig. 7) and discussions during the workshop sessions revealed differences in opinion towards treatment strategies to achieve PsA eradication.

A literature review on eradication studies of Pseudomonas Aeruginosa infection in PCD, cystic fibrosis (CF) and non-CF/PCD-bronchiectasis (Cochrane reviews when available) will be carried out so that possible recommendations can be derived and form the basis for future randomized clinical trials among best-ranked methods or treatments for eradication of PsA infection in PCD. Meanwhile a European survey with the specific aim to aggregate knowledge on various existing treatment regimens regarding PsA infection in PCD is planned with a view to combine results of the review and the survey for intermediate guidelines to prevent or treat such infections.

Effective treatments for sinus diseases and hearing problems need to be identified in order to propose treatment modalities that can be the subject of randomised controlled trials. A recent publication on the clinical effects of sinus surgery and intensive follow-up in patients with PCD revealed bacterial sinusitis in 19/20 patients demonstrating simultaneous sinus and lung infection with identical pathogen in 13/20 patients [28], highlighting the need for further and larger studies to address the possible importance of a more aggressive treatment approach towards the inevitable chronic sinusitis in PCD patients.

An assessment of the risk of cross infection in PCD was discussed. Similar to CF, the PCD community of patients and physicians fears interpersonal transmission of infections; however a previous study investigating cross infection in 107 PCD patients from a single centre during an 11-year follow-up period showed no patient to patient transmission [28]. Guidelines and recommendations on the need for cross infection precautions in outpatient clinics and in relation to patient’s participation in future meetings are necessary.

**Fig. 8:**
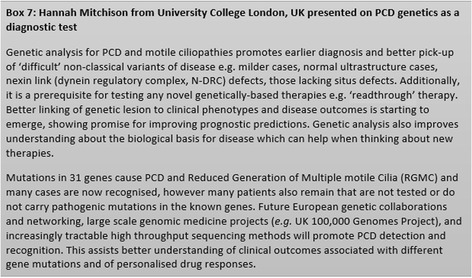
Hannah Mitchison from University College London, UK presented on PCD genetics as a diagnostic test

Reasons for variations in disease severity and progression between patients diagnosed with PCD was an area of interest for WG participants. It is likely that a combination of different factors are responsible for these variations, several of which are being investigated. There has been growing interest in genotype-phenotype correlations [35–37], with studies reporting milder or more severe phenotypes depending on the genes affected [37–40]. The different applications of genetic analysis in PCD was presented (Fig. 8).

A survey investigating differences in delivery of care for PCD across Europe was discussed. Questionnaires and in-depth interviews with PCD specialists from different countries will provide in-depth information for the development of a survey, which will collect data from healthcare professionals involved in PCD diagnostics and management.

## Work group 4: Clinical trials

The clinical trials workgroup (WG4) aims to develop the evidence-base to underpin future clinical trials. It will identify and evaluate clinically relevant outcome measures for longitudinal monitoring of PCD patients in clinical care and future trials.

Major areas of interest for collaboration identified by WG4 include:

- Enhancing the evidence base for disease-targeted treatments;
- Identifying challenges in designing and conducting clinical trials in PCD; and
- Deeper phenotyping of PCD patients for better stratification in clinical trials.

**Fig. 9:**
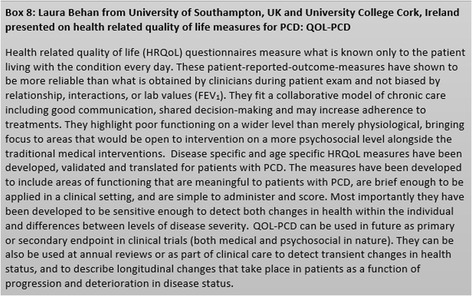
Laura Behan from University of Southamton, UK and University College Cork, Ireland presented on Health related quality of life measures fro PCD: QOL-PCD

Clinically validated outcome measures are essential for the reporting of clinical trials and longitudinal studies [41]. This is particularly important for international collaborations and the expansion of ongoing research networks [42, 43]. Establishing clear disease-defining and monitoring outcomes and algorithms would increase data validity and shareability, and allow for cross-study comparisons. The development, validation, and translation of the first outcome measure in PCD, which assesses health related quality of life questionnaire [20], was presented (Fig. 9).

**Fig. 10:**
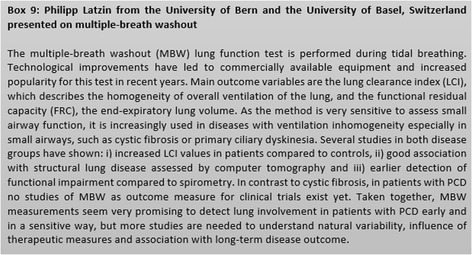
Philipp Latzin from the University of Bern and the University of Basel, Switzerland presented on multiple-breath washout

**Fig. 11:**
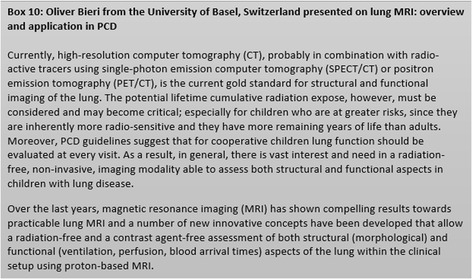
Oliver Bieri from the University of Basel, Switzerland presented on lung MRI: overview and application in PCD

Previous studies in PCD have used a wide range of outcomes as surrogate measures for disease severity. Forced expiratory volume in 1 s as a percentage of predicted (%FEV_1_), for example, is frequently used as an indicator for severity of lung disease despite studies suggesting it might not be appropriate in longitudinal research and clinical trials as a single outcome measure for early lung disease in PCD [44, 45]. Discussions highlighted the necessity of identifying other possible outcome measures for monitoring lung disease progression and to serve as endpoints in clinical trials, such as multiple breath wash-outs (MBW), high-resolution computerised tomography and magnetic resonance imaging. Future applications of MBW and MRI in PCD and as a potential outcome measure for future clinical trials were presented at the conference (Figs. 10 and 11). WG4, thus, aims to review and summarise the existing evidence on the use of outcome measures in PCD. This will result in a series of systematic reviews, which will identify and evaluate disease specific outcome measures to be recommended for future use. Additionally, participants agreed on the importance of conducting a review on outcome measures used in other diseases that share some similarity with PCD, such as cystic fibrosis and chronic rhinosinusitis, to suggest outcomes that might be of relevance but have not yet been used or investigated in PCD. This will result in a positional paper that will inform future research studies.

Little is known about the natural variability of lung function parameters in stable patients over time. Correlation of %FEV_1_ with other lung function measurements such as lung clearance index (LCI) has been investigated, with studies showing conflicting results [46–49]. Furthermore, there are very few studies [50] investigating the precision of individual measurements and no studies investigating repeatability and response to change of respiratory status. Interventions such as physiotherapy and use of mucolytics will likely influence lung function parameters. An ongoing study by Boon et al. is investigating differences in %FEV_1_ and LCI before and after 20 min of chest physiotherapy (ClinicalTrials.gov ID NCT01929356). Participants discussed a framework for an international longitudinal cohort study using prospectively collected routine clinical data to assess the natural variability of lung function parameters in stable PCD patients. The primary objective is to identify minimal variations on lung function parameters, upon intervention, that are clinically relevant and therefore should be taken into consideration when planning clinical trials and collecting data for longitudinal studies. One of the main challenges participants identified was the need to define stability and pulmonary exacerbation. Discussions between WG4 participants revealed that the definition of exacerbation varied considerably among PCD specialists; there is a need for a standardised clinical proforma with a clear definition of pulmonary exacerbation, which was discussed in further details and will be developed in close collaboration with WG2.

**Fig. 12:**
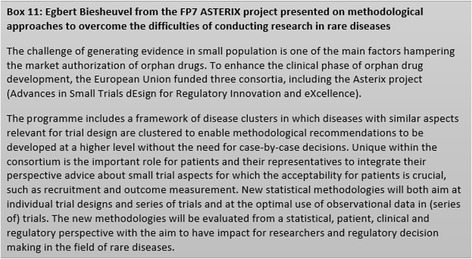
Egbert Biesheuvel from the FP7 ASTERIX project presented on methodological approaches to overcome the difficulties of conducting research in rare diseases

Limitations associated with conducting clinical trials in rare diseases are well known (e.g., limited study population, poor phenotyping) [51, 52]. Underpowered studies are particularly common in PCD; new statistical strategies to deal with methodological challenges derived from small samples are being addressed [53]. During the inaugural conference, participants heard from one of three European consortiums working on developing new statistical methods for rare diseases and small populations (Fig. 12). Collaboration with these statistical networks will provide an additional toolbox from which BEAT-PCD members can draw expertise for new study designs tailored for rare diseases, including the use of Bayesian and adaptive designs. It was also discussed that routinely collected data represent a rich and ample source for research but concerns over data standardisation across different countries should be fully addressed. An important step in this direction was the recent establishment of the European PCD registry [19].

## Vision for training school and short term scientific missions

BEAT-PCD provides an innovative training program for Early Stage Researchers (ESR) which, with the Action’s network of experts, consensus guidelines, standardised protocols and infrastructure, will underpin a global translational research program of PCD led by European researchers. A poster session at the BEAT-PCD Inaugural Conference demonstrated the considerable depth and breadth of research being undertaken by the ESRs within the COST action. Topics ranged from the development of a mathematical model for measurement of cilia motion to the ongoing randomised controlled trial to determine the efficacy and safety of azithromycin maintenance therapy in PCD (Table 1).

**Table 1:** Title, authors and country of first author of 32 posters from 10 countries, presented at the BEAT-PCD Inaugural Conference (only listed if authors agreed to have the title published)

Poster title	Authors (*Country of first author*)
Opportunities and challenges In the development and evaluation of a clinical questionnaire for PCD.	Amirav I, Roberts MR, Mussaffi H, Revital A, National Israeli PCD Consortium, Omran H *(Israel)*
PCD in Israel: prevalence, clinical features, current diagnosis and management practices.	Abitbul R, Amirav I, National Israeli PCD Consortium, Omran H, Mussaffi H *(Israel)*
Diagnosing primary ciliary dyskinesia; an international patient perspective.	Behan L, Galvin AD, Masefield S, Copeland F, Manion M, Rindlisbacher B, Redfern B, Rubbo B, Lucas JS *(UK)*
*Ex-vivo* measurement and mathematical model of a flow induced by cilia motion: application in primary ciliary dyskinesia.	Bottier M, Pena Fernandez M, Blanchon S, Pelle G, Bequignon F, Isabey D, Coste A, Escudier E, Grotberg JB, Papon JF, Louis B, Filoche M *(France)*
Physical parameters of metachronal waves in healthy and diseased human airway epithelium.	Chioccioli M *(UK)*
The accuracy of nasal nitric oxide testing in PCD diagnostics is population specific.	Collins SA, Behan L, Evans HJ, Goggin P, Harris A, Jackson C, Packham S, Walker WT, Lucas JS *(UK)*
Clinical index to evaluate the risk of primary ciliary dyskinesia in children.	Djakow D, Rozehnalova E, Havlisova M, Svobodova T, Pohunek P *(Czech Republic)*
Clinical evaluation of primary ciliary dyskinesia patients.	Emiralioglu N, Ozcelik U, Tugcu GD, Yalcin E, Ersoz DD, Kiper N *(Turkey)*
Comparison of bronchoscopy and sputum findings in patients with primary ciliary dyskinesia.	Emiralioglu N, Sener B, Tugcu GD, Yalcin E, Ersoz DD, Kiper N, Ozcelik U *(Turkey)*
Radiological findings of patients with primary ciliary dyskinesia.	Emiralioglu N, Oguz B, Gunes A, Yalcin E, Ersoz DD, Kiper N, Ozcelik U *(Turkey)*
Therapeutic potential of dynein assembly defects in PCD.	Fassad MR, Shoemark A, Patel M, Hayward J, Boustread C, Jenkins L, Cullup T, Hogg C, Mitchison HM *(UK)*
Clinical characteristics and follow up in adult patients with primary ciliary dyskinesia.	Frija-Masson F, Bassinet L, Honore I, Desmazes-Dufeu N, Housset B, Coste A, Escudier E, Brugel P-R, Maitre B *(France)*
Diagnosis of primary ciliary dyskinesia (PCD) by transmission electron microscopy (TEM); validation of a quantitative method.	Goggin P, Page A, Pickering R, Lucas JS *(UK)*
Growth in patients with primary ciliary dyskinesia (PCD): a multinational study.	Goutaki M, Spycher B, Maurer E, Meier B, Amirav I, Behan L, Boon M, Carr S, Casaulta C, Clement A, Crowley S, Dell S, Ferkol T, Haarman E, Karadag B, Knowles M, Koerner-Rettberg C, Leigh M, Loebinger M, Mazurek H, Morgan L, Nielsen K, Philipsen M, Sagel S, Santamaria F, Scwerk N, Yiallouros P, Werner C, Kuehni C *(Switzerland)*
Lung function in patients with primary ciliary dyskinesia (PCD): a multinational study.	Halbeisen F, Goutaki M, Maurer E, Boon M, Casaulta C, Clement A, Crowley S, Haarman E, Karadag B, Koerner-Rettberg C, Mazurek H, Morgan L, Nielsen KG, Santamaria F, Schwerk N, Yiallouros P, Latzin P, Lucas JS, Kuehni C *(Switzerland)*
Is the portable NIOX MINO reliable for screening nasal nitric oxide levels in primary ciliary dyskinesia?	Harris A, Bhullar E, Joslin R, Gove K, Lucas JS *(UK)*
Ready, Steady, Go (RSG): addressing the needs of adolescents in transition from child to adult services.	Harris A, Maddison J, McGinnity T, Nagra A, Lucas JS *(UK)*
Ciliary abnormalities in primary ciliopathies: are these also classed as PCD patients?	Hirst RA, Rutman A, Williams G, Kulkarni N, O’Callaghan C *(UK)*
Accuracy of primary ciliary dyskinesia diagnostic tests: experience from a national diagnostic centre [54]	Jackson CL, Behan L, Goggin PM, Adam E, Coles JL, Evans H, Harris A, Lackie P, Packham S, Page A, Thompson J, Walker W, Kuehni C, Lucas JS *(UK)*
Evidence for seasonal variation in ciliation of airway epithelial cells cultured at air-liquid interface; samples for primary ciliary dyskinesia testing [55].	Jackson CL, Coles JL, Thompson J, Lucas JS *(UK)*
A novel form of PCD that impacts nodal, but not tracheal cilia.	Keynton J, Adams E, Riley K, Powles-Glover N, Shinohara K, Lucas J, Lackie P, Norris D *(UK)*
Randomized controlled trial to determine the efficacy and safety of azithromycin maintenance therapy in primary ciliary dyskinesia.	Kobbernagel HE, Buchvald FF, Casaulta C, Collins S, Haarman EG, Hogg C, Kuehni C, Lucas JS, Omran H, Werner C, Nielsen KG *(Denmark)*
Electron tomography detects ultrastructural abnormalities in patients with primary ciliary dyskinesia due to defects in DNAH11.	Kwan R, Burgoyne T, Dixon M, Patel M, Scully J, Onoufriadis A, Hogg C, Mitchison HM, Shoemark A *(UK)*
Single breath washout as alternative to multiple breath washout in patients with primary ciliary dyskinesia (PCD)?	Nyilas S, Schlegtendal A, Yammine S, Casaulta C, Latzin P, Koerner-Rettberg C *(Switzerland)*
Physiological phenotyping of paediatric chronic obstructive airway disease [56].	Nyilas S, Singer F, Kumar N, Yammine S, Meier-Girard D, Koerner-Rettberg C, Casaulta C, Frey U, Latzin P *(Switzerland)*
Colonization with Pseudomonas aeruginosa in patients with primary ciliary dyskinesia – a relevant complication?	Price M, Brinkmann G, Hansen G, Schwerk N *(Germany)*
Growing up with primary ciliary dyskinesia in Bradford: exploring patients’ experiences as a physiotherapist.	Schofield LM, Horobin HE *(UK)*
Accuracy of immunofluorescence in the diagnosis of primary ciliary dyskinesia.	Shoemark A, Frost E, Dixon M, Olloson S, Kilpin K, Bush A, Hogg C *(UK)*
Impaired childhood growth velocity in patients with primary ciliary dyskinesia.	Svobodova T, Djakow J, Zemkova D, Cipra A, Pohunek P, Lebl J *(Czech Republic)*
Living with primary ciliary dyskinesia.	Taelman A, Boon M, Dupont L, Havermans T (Belgium)
The unicellular organism *Paramecium* as a model system to study PCD.	Tassin AM *(France)*
*Paramecium tetraurelia* basal body unit isolation for cryo-electron tomography studies.	Trepout S, Lemullois M, Guichard P, Koll F,Fleury AA, Beisoon J, Cohen J, Marco S, Tassin AM *(France)*

Training of ESRs will be conducted through Training Schools (TS) and Short-Term Scientific Missions (STSMs) and will ensure sustainability and development in the field of PCD research. Having trained together from an early stage, it is anticipated that the scientists and clinicians will be positioned to continue moving translational research in PCD forward.

The aim of Training Schools is to widen, broaden and share knowledge relevant to BEAT-PCD’s objectives through the delivery of intensive training on new and emerging subjects. The Training School will establish themes in its inaugural year that can be developed over the following three TS. Apart from the lectures delivered by senior experts in the field, the workshops, poster sessions and short presentations have been designed following surveys to the ESRs to fulfil current training needs. We will gather feedback on the TS and develop ongoing programmes to extend and develop the programme to fit emerging science and clinical advances. Training Schools will provide ESRs with an opportunity to interact and collaborate with senior academics, clinicians, students, postdoctoral fellows and invited speakers from both academia and industry at Conferences, Workshops and Training Schools, thus providing a unique career development opportunity.

The aim of Short-Term Scientific Missions is to support individual mobility, strengthen existing networks, and foster collaborations by allowing ESRs to visit an institution or laboratory in another participating COST Country, an approved NNC institution, or an approved IPC institution. STSMs provide the opportunity for ESRs to learn from experts in the field by providing the necessary resources for BEAT-PCD members to train with other PCD teams and/or invite experts in particular areas and techniques to visit their institution. This exchange of experiences promotes standardisation of procedures across institutions, allows applicants to learn new techniques or gain access to specific instruments and/or methods not available in their own institutions, and provides a platform for career progression for ESRs as well as development of new ideas between established researchers. Approximately ten bursaries will be awarded by competitive applications each year as contributions to the missions.

**Fig. 13:**
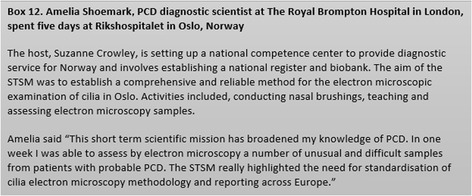
Amelia Shoemark, PCD diagnostic scientist at The Royal Brompton Hospital in London, spent 5 days at Rikshospitalet in Oslo, Norway

**Fig. 14:**
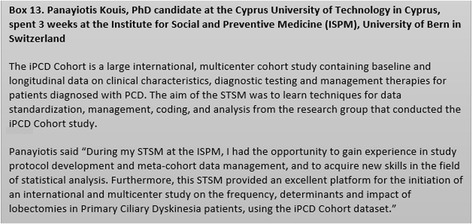
Panayiotis Kouis, PhD candidate at the Cyprus University of Technology in Cyprus, spent 3 weeks at the Institute for Social and Preventive Medicine (ISPM), University of Bern in Switzerland

Examples of opportunities for STSMs are outlined on the BEAT-PCD website (http://www.beatpcd.org/). These include training in PCD diagnostic techniques, systematic reviews and meta-analysis focused on rare diseases, data management and statistical methods for multicentre data analysis, and clinical management for children with PCD (Figs. 13 and 14). Alternatively individuals can identify their own needs and liaise directly with specialist centres to establish a project.

## Summary

The BEAT-PCD Inaugural Conference successfully brought together clinical PCD specialists (paediatricians and adult pulmonologists, ENT, physiotherapists, specialist nurses) and scientists from varied backgrounds (genetics, imaging, cell biology, microbiology, bioinformatics). The multidisciplinary conference provided an interactive platform for research groups from twenty countries to exchange ideas through a program of lectures, poster presentations, breakout sessions and workshops.

**Fig. 15:**
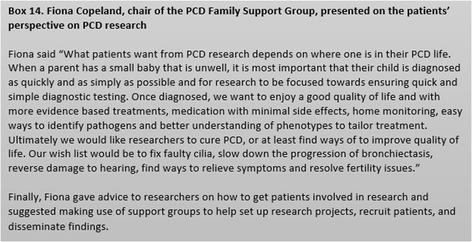
Fiona Copeland, chair of the PCD Family Support Group, presented on the patients’ perspective on PCD research

A series of lectures and posters provided an overview of recent work in the PCD field. Participants heard comprehensive talks on the state-of-the-art diagnosis and management from basic science and clinical perspectives, and on lessons learned from previous research. The patients’ perspectives on PCD research was delivered by the Chair of the UK PCD Family Support Group who highlighted the need for more evidence based treatments, medication with minimal side effects, home monitoring and better understanding of phenotypes for which to tailor treatment (Fig. 15). The workshops and breakout sessions encouraged discussion on gaps in the current knowledge and the need for research focused on patient benefit. The conference provided the ideal environment, bringing together both existing and new potential collaborators from a wide range of disciplines and countries. Ongoing projects were discussed and in parallel, new projects developed. The new studies emerged from both the shared interests of those present as well as from specific needs identified during the meeting.

Additionally, collaborations between ESRs and senior experts in different research groups and across specialties were encouraged throughout the conference. ESRs actively participated by chairing and giving lectures, presenting posters, organising breakout sessions and contributing ideas for future projects.

As outlined, a range of highly collaborative projects were developed as a direct result of this network of experts coming together. COST-Action provides the perfect platform to develop and move these projects forward by enabling networking events such as this one. The proposed projects are essential for scientific advancement and translational research and will lead to the improvement of outcomes for patients with PCD. Although proposed projects are ambitious in size and number, the partnerships formed between research groups will minimise duplication of effort and lost opportunities, therefore increasing the quantity and quality of scientific output. The BEAT-PCD network provides a direct pathway facilitating the wider dissemination of findings across Europe and beyond.
